# Radiometabolic Therapy in Lymphoma: From Radioimmunotherapy to Emerging Theranostic and Combination Strategies

**DOI:** 10.3390/cancers18121960

**Published:** 2026-06-16

**Authors:** Agostino Chiaravalloti, Daniele Di Biagio, Pierpaolo Alongi, Elizabeth Katherine Triumbari, Annalisa Noce, Michele Basilicata, Ferdinando Calabria

**Affiliations:** 1 Department of Biomedicine and Prevention, University of Rome Tor Vergata, 00133 Rome, Italy; 2 IRCCS Neuromed, Via Atinense 18, 86077 Pozzilli, Italy; 3 Centro di Medicina Nucleare, Ospedale San Pietro Fatebenefratelli, 00189 Rome, Italy; d.dibiagio@expressdiagnostic.it; 4 Department of Biomedicine, Neuroscience and Advanced Diagnostics (BiND), University of Palermo, Via del Vespro 129, 90127 Palermo, Italy; pierpaolo.alongi@unipa.it; 5 Santa Maria Goretti Hospital, Via Antonio Canova, 04100 Latina, Italy; 6 Department of Systems Medicine, University of Rome Tor Vergata, 00133 Rome, Italy; 7 UOSD Nephrology and Dialysis, Policlinico Tor Vergata, 00133 Rome, Italy; 8 Department of Clinical Sciences, Catholic University Our Lady of Good Counsel, 1000 Tirana, Albania; 9 UOSD Special Care Dentistry, Department of Experimental Medicine and Surgery, University of Rome Tor Vergata, 00133 Rome, Italy; michele.basilicata@ptvonline.it; 10 Department of Nuclear Medicine and Theragnostics, “Mariano Santo” Cosenza Hospital, Contrada Muoio Piccolo, 87100 Cosenza, Italy; ferdinandocalabria@hotmail.it

**Keywords:** lymphoma, radioimmunotherapy, radioligand therapy, radiometabolic therapy, theranostics, CXCR4, CD20, CD22, CD37, targeted radionuclide therapy

## Abstract

Radiometabolic therapy is a mechanistically plausible but clinically underused strategy in lymphoma. Its rationale is based on the selective delivery of cytotoxic radiation to malignant lymphoid cells through antibodies, peptides, or small molecules directed against tumor-associated targets. Radioimmunotherapy with anti-CD20 agents, including 90Y-ibritumomab tiuxetan and 131I-tositumomab, demonstrated meaningful efficacy in B-cell non-Hodgkin lymphoma, particularly in indolent and relapsed/refractory settings. However, despite encouraging clinical results, its use progressively declined because of logistical, regulatory, commercial, and multidisciplinary barriers. More recently, renewed interest has emerged with the development of novel antibody–radionuclide conjugates and radioligand-based theranostic strategies targeting CD22, CD37, CD45, and CXCR4. Among these, CXCR4-directed imaging and therapy with 68Ga-pentixafor and 177Lu/90Y-pentixather illustrate image-guided patient selection and targeted radionuclide treatment in advanced hematologic malignancies. This narrative review summarizes evidence retrieved from Scopus and PubMed on radiometabolic therapy in lymphoma, with particular attention paid to established radioimmunotherapy, emerging targets, radioligand therapy, dosimetry, toxicity, and combination strategies with chemotherapy, immunotherapy, and hematopoietic stem cell transplantation. Available evidence supports the plausibility and possible clinical utility of these approaches, but remains heterogeneous and, for several newer targets, preliminary. Future development will require prospective trials, standardized imaging-based selection, individualized dosimetry, and integration within multidisciplinary lymphoma treatment pathways.

## Simple Summary

Some lymphoma treatments can carry radiation directly to cancer cells by using antibodies or small molecules that recognize specific targets. Older CD20-directed radioimmunotherapy showed that this strategy can work, particularly in selected B-cell lymphomas, but it has remained difficult to implement in everyday practice. Newer approaches aim to select patients more carefully with molecular imaging, estimate radiation dose to tumors and organs, and integrate treatment with chemotherapy or transplantation when appropriate. This review summarizes what is established, what remains investigational, and why patient selection, dosimetry, safety monitoring, and realistic clinical workflows are essential for future development.

## 1. Introduction

Lymphomas comprise a heterogeneous group of hematologic malignancies with distinct biological behavior, clinical presentation, treatment sensitivity, and prognosis. Non-Hodgkin lymphoma represents the largest and most diverse category, ranging from indolent B-cell neoplasms to aggressive lymphomas requiring intensive systemic therapy [1]. Follicular lymphoma is a paradigmatic setting in which monoclonal antibodies, immunochemotherapy, and post-induction strategies have changed long-term disease control, but relapse, treatment resistance, cumulative toxicity, and limited options for heavily pretreated patients remain clinically relevant problems [2].

Within this evolving scenario, radiometabolic therapy remains an underused therapeutic strategy with a clear mechanistic rationale. In lymphoma, the historical and most widely used term remains radioimmunotherapy, because the first clinically successful approaches used monoclonal antibodies as radionuclide carriers. In this review, the term radiometabolic therapy is used more broadly to include both antibody-based radioimmunotherapy and newer ligand-based theranostic strategies designed to deliver cytotoxic radiation selectively to lymphoma cells or to lymphoma-associated targets. Unlike external beam radiotherapy, systemic radionuclide-based approaches may potentially treat disseminated disease while preserving a degree of biological selectivity through antibody-, peptide-, or small-molecule-mediated targeting [3].

Anti-CD20 radioimmunoconjugates, including 90Y-ibritumomab tiuxetan and 131I-tositumomab, provided the first mature proof of principle that lymphoma can be effectively targeted by systemic radiation delivered through immunologic recognition [4,5,6,7]. Despite this evidence, radioimmunotherapy did not become a widely adopted component of lymphoma treatment. Its decline was likely multifactorial, reflecting logistical complexity, radiation-safety requirements, limited multidisciplinary integration, reimbursement and commercial issues, competition with rapidly expanding non-radioactive systemic therapies, and the lack of contemporary head-to-head comparisons with newer immunologic or targeted treatments [3,8].

More recently, interest in radionuclide-based treatment for lymphoma has been renewed by the broader development of theranostics and radioligand therapy in oncology. Newer approaches extend beyond CD20 and include targets such as CD22, CD37, CD45, and C-X-C chemokine receptor type 4 (CXCR4), with increasing attention paid to imaging-based patient selection, individualized dosimetry, and integration with transplantation or systemic therapies [8]. CXCR4-directed theranostics is particularly relevant because 68Ga-pentixafor PET can non-invasively assess target expression, whereas 177Lu- or 90Y-labeled pentixather has been explored therapeutically in advanced hematologic malignancies, including diffuse large B-cell lymphoma and T-cell lymphoma [9,10]. The current evidence, however, remains heterogeneous. Established anti-CD20 radioimmunotherapy is supported by older clinical trials, whereas several emerging radioligand or next-generation radioimmunotherapy approaches are based on early-phase studies, small cohorts, preclinical data, or mixed hematologic populations. Therefore, the field should not be interpreted as a mature therapeutic standard across lymphoma subtypes, but rather as a biologically plausible and clinically promising area requiring careful reassessment in the modern era of combination treatment.

This review summarizes the available evidence on radiometabolic therapy in lymphoma, focusing on established radioimmunotherapy, emerging radioligand and theranostic approaches, combination strategies, toxicity, dosimetry, implementation barriers, and future perspectives.

## 2. Literature Search Strategy

**Figure 1:**
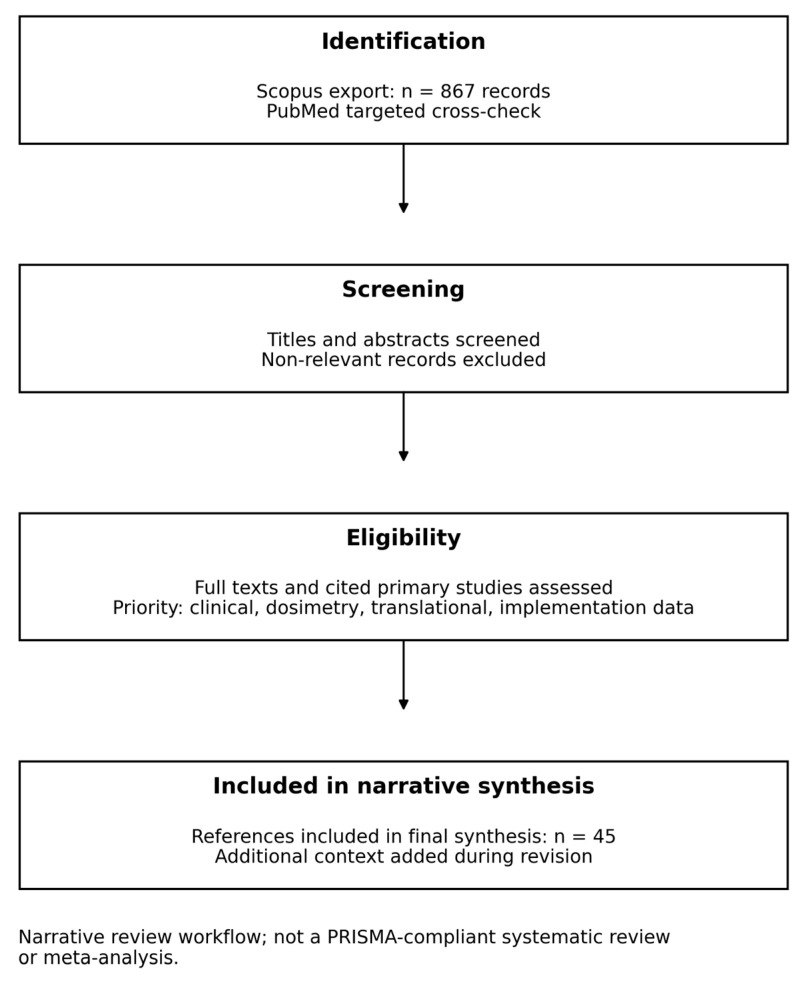
Narrative literature search workflow. The diagram summarizes the Scopus export, PubMed cross-check, screening priorities, and final narrative synthesis. This figure is intended to improve transparency and does not represent a PRISMA-compliant systematic review or meta-analysis.

A narrative literature search was performed in Scopus and PubMed to identify peer-reviewed articles relevant to radiometabolic therapy in lymphoma. The Scopus export used for this revision was generated on 1 May 2026 and contained 867 records; PubMed was cross-checked using the same keyword structure and targeted verification of key records. Search terms included combinations of “lymphoma”, “Hodgkin lymphoma”, “non-Hodgkin lymphoma”, “radioimmunotherapy”, “radioligand therapy”, “targeted radionuclide therapy”, “theranostics”, “ibritumomab”, “tositumomab”, “epratuzumab”, “lilotomab”, “pentixafor”, “pentixather”, “CD20”, “CD22”, “CD37”, “CD45”, and “CXCR4”. Title/abstract screening and final inclusion were performed by two authors, with disagreements resolved by discussion. Priority was given to original clinical studies, early-phase trials, prospective or retrospective clinical series, dosimetry studies, and translational articles directly addressing radionuclide-based treatment or target imaging in lymphoma. Reviews and guidelines were used only when necessary to contextualize implementation, terminology, historical development, or comparison with modern immunotherapies. Articles not specifically related to lymphoma or radionuclide-based therapy, including generic immunotherapy or nanomedicine reports without a radiometabolic component, were excluded from the core synthesis. Because of the heterogeneity of disease subtypes, targets, radionuclides, and treatment settings, the review was structured narratively rather than as a formal systematic review or meta-analysis. The search and selection workflow is summarized in Figure 1.

## 3. Biological Rationale of Radiometabolic Therapy in Lymphoma

The rationale for radiometabolic therapy in lymphoma derives from the convergence of three biological and clinical features: the frequent expression of targetable surface antigens or receptors, the intrinsic radiosensitivity of many lymphoma subtypes, and the systemic nature of the disease. Unlike external beam radiotherapy, which is anatomically restricted, antibody- or ligand-mediated radionuclide delivery may expose multiple disease sites to cytotoxic radiation while retaining a degree of molecular selectivity. This concept is particularly relevant in lymphomas, where malignant cells often express lineage-associated antigens that have already been validated as therapeutic targets by non-radioactive monoclonal antibodies, antibody–drug conjugates, or cellular therapies [2,3].

**Figure 2:**
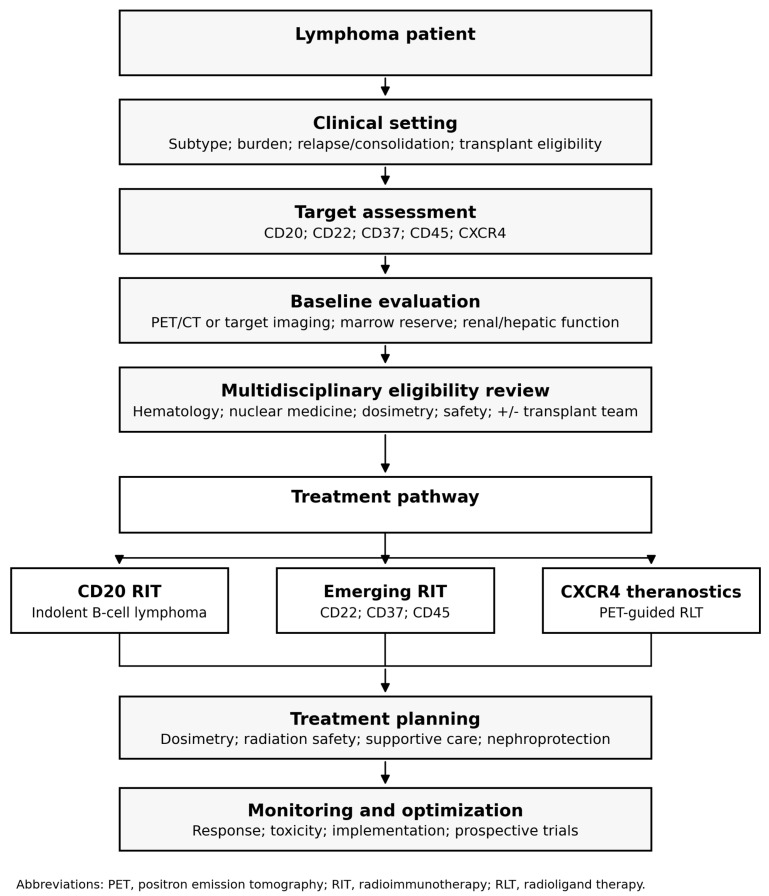
Proposed framework for radiometabolic therapy in lymphoma. The figure summarizes a stepwise approach integrating clinical setting, target assessment, baseline imaging and organ-reserve evaluation, multidisciplinary eligibility review, treatment pathway selection, treatment planning, monitoring, and future optimization. PET, positron emission tomography; RIT, radioimmunotherapy; RLT, radioligand therapy.

Figure 2 provides a schematic framework for clinical decision-making, whereas Table 1 summarizes the main established and emerging radiometabolic therapy strategies in lymphoma. The final column of Table 1 is intended as a guideline-informed interpretation rather than as a formal grade of recommendation. It was assigned using four criteria: (i) whether the strategy is included as a treatment option in contemporary NCCN or ESMO lymphoma recommendations; (ii) regulatory and commercial availability; (iii) maturity of clinical evidence; and (iv) treatment setting, distinguishing routine clinical use from specialized transplant workflows, clinical trials, or compassionate-use experience [11,12].

### 3.1. Target Antigens and Receptors

CD20 represents the historical prototype of a lymphoma-associated target for radioimmunotherapy. It is broadly expressed in mature B-cell lymphomas and has been therapeutically exploited by unconjugated antibodies and by radiolabeled anti-CD20 constructs. The two best-known agents, 90Y-ibritumomab tiuxetan and 131I-tositumomab, both target CD20 and provided the first mature clinical demonstration that monoclonal antibodies can be used not only as immune effector agents, but also as carriers of tumor-directed radiation [4,5]. The biological appeal of CD20-directed radioimmunotherapy is based on target prevalence, favorable tumor-to-background localization, and the ability of the radionuclide payload to induce DNA damage in antigen-expressing lymphoma cells and neighboring malignant cells through a cross-fire effect.

CD22 is another B-cell-associated antigen of interest. It is expressed in several B-cell malignancies and has been explored with radiolabeled epratuzumab-based constructs. Compared with CD20, CD22-directed strategies offer a partly distinct targeting axis and may be relevant in settings where prior anti-CD20 exposure, antigen modulation, or resistance to conventional immunochemotherapy limits therapeutic options. Fractionated anti-CD22 radioimmunotherapy has been investigated in non-Hodgkin lymphoma, including clinical studies with 90Y-epratuzumab tetraxetan [20,21].

CD37 has also emerged as a relevant target in B-cell lymphomas. Its expression across mature B-cell neoplasms, together with its relatively restricted distribution in normal tissues, provides a rationale for antibody–radionuclide conjugates. Early anti-CD37 radioimmunotherapy experience dates back several decades, but interest has been renewed by the development of 177Lu-lilotomab satetraxetan, which has been evaluated in indolent non-Hodgkin lymphoma with specific attention paid to biodistribution, dosimetry, and hematologic toxicity [24,35].

CXCR4 differs from B-cell lineage antigens. It is a chemokine receptor involved in tumor cell trafficking, homing, bone marrow interaction, and microenvironmental retention. In hematologic malignancies, the CXCL12–CXCR4 axis contributes to the interaction between malignant cells and protective stromal niches. This makes CXCR4 both a marker of disease biology and a therapeutic target. CXCR4-directed theranostics is particularly relevant because 68Ga-pentixafor PET can evaluate receptor expression in vivo, while 177Lu- or 90Y-pentixather can deliver therapeutic radiation to CXCR4-expressing disease sites [9,10,28]. This imaging-therapy pair provides a theranostic model distinct from historical radioimmunotherapy, because patient selection, disease mapping, and treatment planning may be informed by target expression on diagnostic PET.

### 3.2. Advantages over Unconjugated Immunotherapy

Radiometabolic therapy differs from unconjugated immunotherapy because its cytotoxic effect is not exclusively dependent on immune effector mechanisms. Conventional monoclonal antibodies act through mechanisms such as antibody-dependent cellular cytotoxicity, complement activation, direct signaling effects, and immune modulation. These mechanisms may be impaired by tumor immune escape, antigen density, prior antibody exposure, microenvironmental protection, or reduced immune effector function in heavily pretreated patients. By contrast, radioimmunotherapy and radioligand therapy add a physical cytotoxic mechanism: localized ionizing radiation.

This distinction is important in lymphoma. Radiation-induced DNA damage can kill targeted cells even when immune-mediated killing is inefficient. In addition, beta-emitting radionuclides such as ^90^Y and ^177^Lu produce a cross-fire effect, whereby radiation emitted from bound cells can damage adjacent malignant cells that may express lower or heterogeneous levels of the target antigen. This may partially overcome intratumoral antigen heterogeneity, a relevant limitation of purely antibody-mediated strategies. At the same time, cross-fire is a double-edged mechanism: it may increase antitumor efficacy, but it can also contribute to off-target toxicity, especially bone marrow toxicity, depending on disease distribution, tumor burden, marrow involvement, prior therapies, radionuclide properties, and administered activity.

Radiometabolic therapy also creates opportunities for rational combinations. It can be combined with chemotherapy, anti-CD20 antibodies, hematopoietic stem cell transplantation, or potentially immune-modulating agents. In this context, the radionuclide component may act as a cytotoxic intensifier, while the antibody or ligand provides tumor localization. However, combination approaches require careful attention to be paid to cumulative hematologic toxicity, timing, patient selection, and dosimetry. Therefore, the theoretical advantage over unconjugated immunotherapy should not be interpreted as automatic superiority, but as a distinct mechanism that may be useful in selected biological and clinical contexts.

### 3.3. Role of the Tumor Microenvironment

The lymphoma microenvironment is not a passive compartment. Stromal cells, immune cells, cytokines, chemokines, extracellular matrix components, and vascular features can influence tumor cell survival, trafficking, immune escape, and treatment resistance. This is particularly relevant for targets such as CXCR4, which links lymphoma cells to marrow and stromal niches through the CXCL12–CXCR4 axis. These interactions may contribute to disease persistence and resistance to systemic therapy, but also create a targetable biological dependency.

From a radiometabolic perspective, the microenvironment has at least three implications. First, target expression is not only a static immunophenotypic feature but may reflect interactions between malignant cells and their tissue niches. Second, radionuclide therapy can affect both tumor cells and adjacent supportive compartments through cross-fire radiation, potentially disrupting protective microenvironmental interactions. Third, target imaging may capture spatial heterogeneity across nodal, extranodal, and marrow sites, allowing a more individualized selection of patients for radioligand therapy.

CXCR4-directed therapy is the clearest example of this concept. 68Ga-pentixafor PET may identify CXCR4-expressing disease and support selection for 177Lu/90Y-pentixather therapy. In this model, radiometabolic therapy is not simply an alternative way to deliver radiation, but a strategy that connects target biology, whole-body molecular imaging, dosimetry, and systemic treatment. This is relevant in advanced lymphoma, where disease is often disseminated and biologically heterogeneous.

Overall, the biological rationale for radiometabolic therapy in lymphoma is substantial, but not uniform across all subtypes or targets. CD20-directed radioimmunotherapy is supported by the most mature clinical experience, whereas CD22, CD37, CD45, and CXCR4-directed approaches represent more specialized or emerging strategies. The next sections therefore critically examine the clinical evidence supporting these approaches, distinguishing established data from preliminary or exploratory results.

### 3.4. Multi-Omics and Precision-Theranostic Patient Selection

Target expression and lesion uptake are necessary but insufficient for optimal patient selection. Modern lymphoma biology increasingly distinguishes molecular subtypes, pathway dependencies, immune-microenvironmental states, and clonal evolution patterns that may influence response to both systemic therapy and radiation-based strategies. In diffuse large B-cell lymphoma, gene-expression and genomic studies have identified molecular and stromal signatures associated with distinct biology and outcomes [36,37,38]. These data suggest that future radiometabolic trials should not rely only on antigen expression or PET positivity, but should also consider whether target expression reflects an actionable tumor dependency, a microenvironmental interaction, or a nonspecific marker of disease burden.

Several examples are relevant. CXCR4-directed imaging may be strengthened by transcriptomic or pathway-based evidence of CXCL12-CXCR4 activation, stromal retention, or marrow-niche dependence. In aggressive B-cell lymphomas, molecular subtypes characterized by B-cell receptor signaling, NF-kappaB activation, immune escape, or stromal signatures could influence whether radiometabolic therapy is likely to act as cytoreduction, consolidation, or conditioning. Spatial biology may further help determine whether target-positive cells are distributed homogeneously, clustered in protective niches, or surrounded by radiosensitive immune and stromal compartments. Radiogenomic correlations could also test whether baseline PET uptake, lesion heterogeneity, and absorbed dose predict response differently across molecular subtypes.

**Figure 3:**
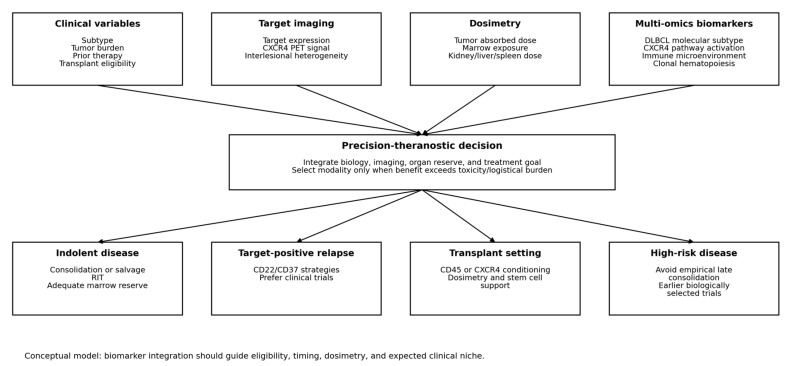
Precision-theranostic integration framework. Clinical variables, target imaging, dosimetry, and multi-omics biomarkers may jointly refine eligibility, timing, treatment allocation, and expected clinical niche for radiometabolic therapy in lymphoma.

A practical precision-theranostic workflow should therefore integrate clinical variables, target-expression imaging, dosimetry, marrow and renal reserve, prior therapies, and multi-omics biomarkers. This does not imply that all patients currently require multi-omics testing before RIT or RLT. Rather, it defines a research agenda for prospective trials and biomarker-enriched cohorts. Figure 3 summarizes this proposed integration framework.

## 4. Established Radioimmunotherapy

Anti-CD20 radioimmunotherapy represents the most extensively investigated form of radiometabolic therapy in lymphoma. The two clinically approved agents that defined this field were 90Y-ibritumomab tiuxetan and 131I-tositumomab, both designed to exploit CD20 expression in mature B-cell lymphomas [3,8]. Their development also illustrates the general requirements for lymphoma radioimmunotherapy: a target antigen expressed at high density on malignant cells, limited expression in critical normal tissues, a carrier with favorable tumor-to-background localization, and a radionuclide whose emission range and half-life are compatible with lymphoma burden, antibody kinetics, and marrow tolerance. Their clinical development provided proof of principle that antibody-mediated radionuclide delivery can induce meaningful and sometimes durable responses in indolent B-cell lymphomas. Representative anti-CD20 trials and consolidation studies are summarized in Table 2.

In relapsed or refractory low-grade, follicular, or transformed B-cell non-Hodgkin lymphoma, 90Y-ibritumomab tiuxetan was directly compared with rituximab in a randomized phase III trial including 143 patients. The overall response rate was significantly higher with 90Y-ibritumomab tiuxetan than with rituximab alone, 80% versus 56%, respectively, and complete responses were also more frequent, 30% versus 16%. However, median duration of response and time to progression were not significantly different between the two arms, indicating that the main advantage of radioimmunotherapy in this study was an increased depth and frequency of response rather than a clear prolongation of disease control in the overall population [13]. In a separate study specifically enrolling patients with rituximab-refractory follicular lymphoma, 90Y-ibritumomab tiuxetan produced an overall response rate of 74%, with 15% complete responses and 59% partial responses. Median time to progression was 6.8 months in the whole cohort and 8.7 months among responders. Toxicity was predominantly hematologic, with grade 4 neutropenia, thrombocytopenia, and anemia reported in 35%, 9%, and 4% of patients, respectively [4].

The corresponding evidence for ^131^I-tositumomab also supported clinically relevant activity in heavily pretreated disease. In the pivotal study of chemotherapy-refractory low-grade or transformed low-grade B-cell non-Hodgkin lymphoma, 60 patients who had received a median of four prior chemotherapy regimens were treated with a single course of ^131^I-tositumomab. The response rate after radioimmunotherapy was 65%, compared with 28% after the patients’ last qualifying chemotherapy regimen. Complete responses occurred in 20% of patients after ^131^I-tositumomab versus 3% after the last chemotherapy regimen. Median duration of response was also longer after radioimmunotherapy, 6.5 versus 3.4 months, while the median duration of complete response had not been reached after more than 47 months of follow-up. Myelodysplasia was reported in four patients during follow-up [5].

131I-tositumomab was also evaluated in patients with B-cell lymphoma progressing after rituximab. In this prospective phase II study, the overall response rate was 65% and the complete response rate was 38%. Median progression-free survival was 10.4 months in the whole cohort, 24.5 months among responders, and was not reached among complete responders. In the subgroup of patients with grade 1–2 follicular lymphoma and tumor size ≤ 7 cm, response appeared more favorable, with overall and complete response rates of 86% and 57%, respectively, and an estimated 3-year progression-free survival of 48%. These findings suggest that tumor burden and histology were important determinants of benefit. Transient grade 3–4 marrow toxicity occurred in approximately half of patients, and secondary myelodysplasia was reported in two patients [14].

Radioimmunotherapy was not restricted to relapsed or refractory settings. As initial treatment for advanced-stage follicular lymphoma, 131I-tositumomab produced high response rates in a single-arm phase II study of 76 previously untreated patients. The overall response rate was 95%, with complete responses in 75% of patients. Molecular responses were documented in 80% of assessable patients with clinical complete response. After a median follow-up of 5.1 years, actuarial 5-year progression-free survival was 59%, and median progression-free survival was 6.1 years. Hematologic toxicity was moderate, no transfusions or hematopoietic growth factors were required, and no cases of myelodysplastic syndrome were observed during the reported follow-up [15]. Although uncontrolled, this study remains important because it showed that a single short course of radioimmunotherapy could induce prolonged remissions in untreated follicular lymphoma.

The strongest randomized evidence for 90Y-ibritumomab tiuxetan in the first-line setting comes from the First-Line Indolent Trial. In the long-term update, 409 patients with advanced follicular lymphoma in complete response, unconfirmed complete response, or partial response after first-line induction were analyzed. Consolidation with 90Y-ibritumomab tiuxetan significantly improved progression-free survival compared with no further treatment. Estimated 8-year progression-free survival was 41% with 90Y-ibritumomab tiuxetan versus 22% in the control arm, with a hazard ratio of 0.47. Median progression-free survival was 4.1 versus 1.1 years, respectively. Median time to next treatment was also substantially longer with radioimmunotherapy, 8.1 versus 3.0 years. However, overall survival was not significantly different between arms. The annualized incidence of myelodysplastic syndrome or acute myeloid leukemia was higher in the 90Y-ibritumomab arm than in the control arm, 0.50% versus 0.07%, respectively [7]. These data demonstrate a durable disease-control advantage, but not an overall survival advantage.

More recent data provide a more nuanced view of anti-CD20 radioimmunotherapy. Rieger et al. reported extended follow-up of 90Y-ibritumomab tiuxetan as first-line treatment in 59 patients aged ≥50 years with follicular lymphoma grade 1–3A. After a median follow-up of 9.6 years, median progression-free survival was 3.6 years, 8-year progression-free survival was 38.3%, median overall survival was not reached, and 8-year overall survival was 69.2%. Importantly, no new safety signals emerged during long-term follow-up, including no apparent increase in secondary malignancies or transformation compared with studies of similar duration [16]. These findings support long-term clinical activity in selected patients, but also show that single-agent radioimmunotherapy is unlikely to replace modern first-line immunochemotherapy or antibody maintenance strategies in unselected follicular lymphoma.

Real-world data are also available. Alhaj Moustafa et al. reported a retrospective Mayo Clinic experience including 51 previously untreated patients with low-grade follicular lymphoma or marginal zone lymphoma treated with standard-dose 90Y-ibritumomab tiuxetan as single-agent first-line therapy. The overall response rate was 100%, with a complete response rate of 94%. Continuous complete response was observed in 59% of patients with more than two years of follow-up, and long-term complete response beyond seven years was observed in 25%. Median progression-free survival was not reached in the whole cohort. However, bulky disease was associated with shorter progression-free survival, 3.5 years versus not reached in non-bulky disease. Grade ≥ 3 thrombocytopenia, neutropenia, and anemia occurred in 47%, 37%, and 4% of patients, respectively, while no therapy-related myelodysplastic syndrome or acute myeloid leukemia was observed [17]. This study reinforces the potential value of radioimmunotherapy in low-tumor-burden indolent lymphoma, but its retrospective design and selection bias must be acknowledged.

The comparison with modern maintenance strategies is less favorable. López-Guillermo et al. reported long-term results of a randomized phase II trial comparing a single dose of 90Y-ibritumomab tiuxetan consolidation with rituximab maintenance for two years in patients with newly diagnosed follicular lymphoma responding to R-CHOP. After a median follow-up of 10.55 years, 10-year progression-free survival was 50% with 90Y-ibritumomab tiuxetan versus 56% with rituximab maintenance, without a significant difference. Overall survival was also not significantly different, with 10-year rates of 78% and 84.5%, respectively. However, the cumulative incidence of second neoplasms at 10 years was higher in the 90Y-ibritumomab arm, 18.5% versus 2% [18]. These results are important because they directly compare radioimmunotherapy consolidation with a clinically established non-radioactive post-induction strategy.

In relapsed follicular lymphoma, radioimmunotherapy has also been explored after cytoreduction. Puvvada et al. evaluated two cycles of ESHAP followed by 90Y-ibritumomab tiuxetan in 28 patients with relapsed follicular lymphoma. The overall response rate was 72%, with complete responses in 45%. However, the study did not meet its primary endpoint: 1-year progression-free survival was 38%, and median progression-free survival was only 10 months, despite median overall survival not being reached. The authors suggested that low accrual, high-risk disease, and insufficient debulking may have contributed to the limited progression-free survival benefit [19]. This study is a useful counterbalance to more favorable first-line or low-burden experiences, showing that radioimmunotherapy is not uniformly effective across all relapsed settings.

Taken together, established anti-CD20 radioimmunotherapy produced high response rates and durable remissions in a subset of patients with indolent B-cell lymphoma, particularly follicular lymphoma and selected marginal zone lymphoma. The most consistent advantages were increased response depth, conversion from partial to complete response, prolongation of progression-free survival, and longer time to next treatment in consolidation settings. However, overall survival benefit was not consistently demonstrated, and efficacy appeared dependent on tumor burden, histology, prior treatment, marrow reserve, and disease chemosensitivity.

The limitations of Zevalin and Bexxar were therefore not merely biological, and their declining use should not be interpreted as a simple failure of antitumor efficacy. Hematologic toxicity was expected and usually reversible, but it remained clinically relevant, especially in heavily pretreated patients or those with marrow involvement. Concerns about secondary myeloid neoplasms were not uniform across studies but were important in long-term analyses and in comparisons with rituximab maintenance. In addition, implementation barriers substantially limited routine adoption: treatment required coordination among hematologists, nuclear medicine physicians, radiopharmacists, physicists, and radiation-safety personnel; 131I-tositumomab required patient-specific dosimetry and thyroid blockade; one anti-CD20 agent was commercially withdrawn; and both agents faced limited availability, reimbursement, regulatory, and commercial challenges [3,8]. Finally, radioimmunotherapy lost clinical visibility during the expansion of non-radioactive immunotherapy and targeted therapy, even though direct contemporary comparisons with many newer treatment classes are largely unavailable. Consequently, despite strong proof of principle, anti-CD20 radioimmunotherapy became progressively underused. This historical experience is central for interpreting newer radioligand and theranostic strategies in lymphoma.

## 5. Emerging Targets and Radioligands

Beyond CD20-directed radioimmunotherapy, several alternative targets have been explored to overcome resistance to anti-CD20-based approaches, improve tumor delivery, and extend radionuclide-based treatment to biologically distinct lymphoma subtypes. The most relevant emerging targets include CD22, CD37, CD45, and CXCR4. These targets differ substantially in biological rationale, clinical maturity, therapeutic setting, and feasibility of theranostic implementation. These approaches are summarized in Table 1.

CD22 is one of the most extensively investigated non-CD20 targets in lymphoma radioimmunotherapy. It is a B-cell-restricted antigen expressed across several B-cell non-Hodgkin lymphoma subtypes and is rapidly internalized after antibody binding. This feature provides a specific rationale for residualizing radiometals such as 90Y, because intracellular retention may improve radiation delivery after receptor-mediated internalization. Early clinical experience with dose-fractionated 90Y-epratuzumab tetraxetan showed that repeated administration was feasible and biologically active. In a single-center phase I/II study including 16 patients with B-cell lymphoma, weekly 90Y-epratuzumab produced an overall objective response rate of 62%, with responses observed in both indolent and aggressive lymphoma. Complete or unconfirmed complete responses occurred in 25% of patients, and durable complete responses were reported. Importantly, response appeared related to CD22 expression: seven of eight patients with strongly positive CD22 expression had objective responses, compared with one of five patients with negative or weak expression [20].

The subsequent multicenter phase I/II study of fractionated 90Y-epratuzumab tetraxetan enrolled 64 patients with relapsed or refractory non-Hodgkin lymphoma, including patients with prior autologous stem cell transplantation. Among 61 evaluable patients, the overall response rate was 62%, with complete or unconfirmed complete responses in 48% and a median progression-free survival of 9.5 months. In patients without prior autologous transplantation, the overall response rate was 71%, with complete or unconfirmed complete responses in 55%. The most favorable results were observed in follicular lymphoma: at the highest total 90Y dose levels, the overall response rate reached 100%, with complete or unconfirmed complete responses in 92% and a median progression-free survival of 24.6 months. In follicular lymphoma refractory to prior anti-CD20-containing regimens, objective and complete/unconfirmed complete responses were both reported in 90% of patients, with a median progression-free survival of 21.5 months [21]. These findings support CD22 as a clinically meaningful target, although the evidence remains based on early-phase, non-randomized experience.

CD22-directed radioimmunotherapy has also been investigated as consolidation after frontline immunochemotherapy. In a prospective, single-group phase II trial in elderly patients with untreated diffuse large B-cell lymphoma, 71 patients received R-CHOP induction and 57 responders subsequently received two weekly doses of 90Y-epratuzumab tetraxetan. With a median follow-up of 37 months, the estimated 2-year event-free survival was 75%. Response status improved after radioimmunotherapy: by CT, complete or unconfirmed complete response increased from 70% after six cycles of R-CHOP to 77% after radioimmunotherapy, whereas PET complete response increased from 56% after R-CHOP to 68% after radioimmunotherapy. However, toxicity was substantial. Grade 3–4 thrombocytopenia occurred in 84% of patients receiving radioimmunotherapy and grade 3–4 neutropenia in 79%. One patient developed myelodysplastic syndrome and one developed acute myeloid leukemia after radioimmunotherapy [22]. Therefore, although anti-CD22 consolidation is conceptually attractive, especially after rituximab-based induction, its hematologic toxicity and lack of randomized comparative validation limit immediate clinical translation.

More recently, CD22 has also been explored as a target for alpha-particle therapy. BAY 1862864, a 227Th-labeled anti-CD22 antibody, was tested in a first-in-human phase I study in patients with relapsed or refractory CD22-positive B-cell non-Hodgkin lymphoma. Twenty-one patients received treatment. The maximum tolerated dose was not reached; one patient in the 4.6 MBq cohort experienced two dose-limiting toxicities, consisting of grade 3 febrile neutropenia and grade 4 thrombocytopenia. Grade ≥ 3 treatment-emergent adverse events occurred in 48% of patients, most commonly neutropenia, thrombocytopenia, and leukopenia. The objective response rate was 25%, including one complete response and four partial responses [23]. These early results show feasibility and tolerability, but antitumor activity was modest. At present, targeted alpha therapy against CD22 should therefore be considered investigational rather than clinically established.

CD37 represents another relevant B-cell target. It is expressed by normal mature B cells and many B-cell lymphomas, while being absent from plasma cells and normal hematopoietic stem cells. This expression pattern makes CD37 attractive for patients previously exposed to CD20-directed therapy. Earlier clinical work with radiolabeled anti-CD37 antibodies provided proof of principle [35], but modern development has focused mainly on 177Lu-lilotomab satetraxetan, a CD37-directed antibody–radionuclide conjugate.

In a phase I/IIa study, 177Lu-lilotomab satetraxetan was evaluated in 74 patients with relapsed or refractory indolent non-Hodgkin lymphoma, including 57 patients with follicular lymphoma. Treatment consisted of a single administration of 177Lu-lilotomab satetraxetan preceded by rituximab and, in selected regimens, unlabeled lilotomab predosing to improve biodistribution. The overall response rate was 61% in the whole cohort and 65% in follicular lymphoma, with complete responses in 30%. Among patients with follicular lymphoma and at least two prior lines of therapy, the overall response rate was 70%, with complete responses in 32%. In rituximab-refractory follicular lymphoma after at least two prior therapies, the overall response rate was 67% and the complete response rate was 24%. Median duration of response was 13.6 months overall and 32.0 months among complete responders. The main toxicity was hematologic, with reversible grade 3–4 neutropenia in 31.6% and thrombocytopenia in 26.3% of patients [25]. These results are clinically relevant because they suggest that CD37-directed radioimmunotherapy can retain activity in relapsed or refractory indolent lymphoma, including rituximab-refractory disease.

Dosimetry studies further support the biological plausibility of 177Lu-lilotomab satetraxetan. Biodistribution analyses showed that, apart from red marrow and tumor, the liver, spleen, and kidneys were the main organs with distinct uptake, with absorbed doses considered modest compared with commonly assumed tolerance limits [24]. A subsequent dosimetric analysis showed that red marrow is the primary dose-limiting organ and that predosing with unlabeled lilotomab significantly improves the tumor-to-red-marrow absorbed dose ratio. Mean tumor-to-red-marrow dose ratios were higher with lilotomab predosing than without predosing, supporting the continued use of predosing strategies to optimize biodistribution [26]. This is an important practical point: for antibody–radionuclide conjugates, clinical activity depends not only on target expression but also on how cold antibody, circulating antigen, marrow exposure, and tumor burden shape therapeutic index.

CD45 is conceptually different from CD22 and CD37. Rather than being a lineage-specific B-cell antigen, CD45 is a panhematopoietic antigen expressed on most nucleated hematopoietic cells. This makes CD45 unsuitable for conventional non-myeloablative lymphoma radioimmunotherapy, but attractive for conditioning strategies before hematopoietic stem cell transplantation. A phase I study evaluated a CD45-targeted antibody–radionuclide conjugate using 131I-labeled BC8 followed by autologous stem cell support in 16 adults with relapsed, refractory, or high-risk lymphoma, including B-cell non-Hodgkin lymphoma, T-cell non-Hodgkin lymphoma, and Hodgkin lymphoma. Absorbed doses up to 32 Gy to the liver were delivered, and no dose-limiting toxicities occurred. Non-hematologic toxicity was infrequent and manageable. Among 14 patients with measurable disease at enrollment, 57% achieved complete remission, including all three patients with T-cell non-Hodgkin lymphoma. Three patients with B-cell non-Hodgkin lymphoma treated at the highest dose levels remained in complete remission without subsequent therapy after 35–41 months [27]. These results are provocative, but CD45-directed therapy should be viewed as a transplantation-associated conditioning approach rather than a broadly applicable standalone lymphoma treatment.

Among the emerging targets, CXCR4 provides the most developed theranostic model. CXCR4 is involved in lymphoma cell trafficking, marrow homing, stromal interactions, and microenvironment-mediated resistance. Unlike antibody-based targets, CXCR4 can be imaged with 68Ga-pentixafor and treated with 177Lu- or 90Y-pentixather. This creates a direct imaging-therapy pair in which PET can be used to assess target expression, map disease distribution, support patient selection, and inform dosimetry before treatment.

Several imaging studies support the relevance of CXCR4-targeted PET in lymphoma. In mantle cell lymphoma, a prospective PET/MRI study compared 68Ga-pentixafor with 18F-FDG in 22 patients. 68Ga-pentixafor PET showed significantly higher sensitivity than 18F-FDG PET, 100% versus 75.2%, while positive predictive value was similar, 94.0% versus 96.5%. Standardized uptake values and tumor-to-background ratios were also significantly higher with 68Ga-pentixafor. For bone marrow involvement, 68Ga-pentixafor SUVmean achieved an area under the curve of 0.92, and for splenic involvement, blood-pool tumor-to-background ratio achieved an area under the curve of 0.81 [29]. These data suggest that CXCR4 PET may be particularly useful in lymphoma subtypes in which FDG uptake is variable or marrow assessment is challenging.

Similar findings have been reported in Waldenström macroglobulinemia/lymphoplasmacytic lymphoma. In a prospective study of 17 patients undergoing both 68Ga-pentixafor PET/CT and 18F-FDG PET/CT, the positive scan rate was higher with 68Ga-pentixafor than with 18F-FDG, 100% versus 58.8%. For bone marrow involvement, sensitivities were 94.1% and 58.8%, respectively. For lymph node involvement, 68Ga-pentixafor was significantly more frequently positive than 18F-FDG, 76.5% versus 11.8%. 68Ga-pentixafor also detected paramedullary and central nervous system involvement not identified by 18F-FDG [30]. In gastric mucosa-associated lymphoid tissue lymphoma after Helicobacter pylori eradication, 68Ga-pentixafor PET/MRI showed high accuracy for detecting residual disease, with pooled accuracy, sensitivity, specificity, positive predictive value, and negative predictive value of 97.0%, 95.0%, 100.0%, 100.0%, and 92.9%, respectively [31]. These imaging studies do not prove therapeutic efficacy, but they strengthen the rationale for CXCR4 as a targetable lymphoma biology axis.

CXCR4-directed radioligand therapy has been explored mainly in advanced, heavily pretreated hematologic malignancies, often as part of conditioning before stem cell transplantation. In advanced diffuse large B-cell lymphoma, six heavily pretreated patients underwent CXCR4-directed radioligand therapy in combination with conditioning chemotherapy and allogeneic stem cell transplantation. All patients had CXCR4 expression confirmed by ^68^Ga-pentixafor PET/CT. Therapy was feasible and acute infusion-related toxicity was not observed. Engraftment occurred after a median of 11 days. However, outcomes were limited: two patients died early from infectious complications, and among four evaluable patients, two partial responses and two mixed responses were reported. Median progression-free survival was 62 days and median overall survival was 76 days [9]. These data demonstrate feasibility but also show that, in very advanced diffuse large B-cell lymphoma, CXCR4-directed radioligand therapy alone is unlikely to overcome aggressive disease biology.

The experience in T-cell lymphoma appears more encouraging, although based on very small numbers. In a series of four patients with advanced, heavily pretreated relapsed T-cell lymphoma, CXCR4-targeted radioligand therapy was used as conditioning before allogeneic or autologous stem cell transplantation. Pretherapeutic dosimetry indicated lymphoma-absorbed doses up to 33.2 Gy. One patient developed tumor lysis syndrome with transient grade 3 kidney failure, and one patient died from septicemia before engraftment; engraftment was achieved in the remaining three patients. During follow-up, one partial response and two complete metabolic responses were observed. Median progression-free survival was 7 months, and after a median follow-up of 54 months, three patients were alive [10]. Although these data are preliminary, they suggest that CXCR4-targeted therapy may have relevant antitumor activity in selected T-cell lymphoma cases.

Dosimetry is central to CXCR4-directed therapy. A biokinetic and dosimetric analysis of 177Lu-pentixather and estimated 90Y-pentixather dosimetry in 19 patients with hematologic neoplasms showed activity retention mainly in kidneys, liver, spleen, and bone marrow. Estimated tumor and extramedullary lesion absorbed doses ranged from 1.5 to 18.2 Gy/GBq of 90Y-pentixather. In non-myeloablative settings, red marrow may be dose-limiting; when stem cell support is planned and myeloablation is intended, kidney dose becomes a major limiting factor [32]. These considerations explain why most clinical CXCR4-directed therapeutic experiences have been embedded within transplantation workflows rather than used as routine outpatient radioligand therapy.

Finally, ligand optimization may further improve the CXCR4 theranostic platform. Second-generation pentixafor/pentixather-based analogs have been developed to improve receptor affinity, cellular uptake, and tumor retention. In preclinical work using Daudi B-cell lymphoma xenografts, selected analogues showed approximately 10-fold higher CXCR4 affinity than reference pentixafor/pentixather compounds, fourfold higher cellular uptake in CXCR4-expressing cells, and improved tumor uptake and retention for 177Lu-labeled analogues [33]. These preclinical data indicate that the current CXCR4 platform remains open to further optimization.

Overall, emerging targets and radioligands in lymphoma show a heterogeneous level of maturity. CD22 and CD37 have the strongest antibody–radionuclide clinical data beyond CD20, with meaningful response rates but relevant hematologic toxicity. CD45 is promising mainly as a transplantation-conditioning strategy. CXCR4 is the most developed theranostic approach, with a solid imaging rationale and early therapeutic feasibility, but clinical evidence remains limited to small, highly selected cohorts. The field therefore supports further development, but not broad clinical adoption outside specialized centers or trials.

## 6. Combination Strategies

Combination strategies represent one of the most clinically relevant directions for radiometabolic therapy in lymphoma. Rather than being used only as isolated salvage treatment, radioimmunotherapy and radioligand therapy have been explored as consolidation after chemotherapy or immunochemotherapy, as part of transplantation-conditioning regimens, and, more recently, as potential partners for immune-based approaches. The main rationale is to exploit the radiosensitivity of lymphoma while using systemic or immune-directed therapies to reduce tumor burden, improve disease control, or support engraftment in high-risk settings. The principal strategies are summarized in Table 1.

In the current lymphoma treatment landscape, radiometabolic therapy should be positioned relative to bispecific antibodies and chimeric antigen receptor T-cell therapies rather than discussed in isolation. These immune-effector approaches have transformed relapsed or refractory B-cell lymphoma management, but they depend on viable immune effector mechanisms, are associated with specific inflammatory toxicities, and may be limited by antigen escape, manufacturing or access constraints, patient frailty, or rapidly progressive disease [39,40]. Radiometabolic therapy has a different mechanism: it delivers physical radiation to target-positive lesions and can exert cross-fire effects on adjacent malignant cells and parts of the microenvironment. Its plausible niche is therefore not to replace cellular or bispecific immunotherapy, but to complement them in selected contexts: low-burden chemosensitive disease, post-induction consolidation, patients requiring cytoreduction before transplantation, or target-positive disease in which imaging and dosimetry indicate a favorable therapeutic index.

This distinction is clinically important. CAR-T cells and bispecific antibodies are dynamic immune therapies; RIT and RLT are spatially and dosimetrically definable radiation therapies. The latter may be attractive when a short-course, non-cellular, imaging-selected treatment is preferable, but it is disadvantaged when marrow reserve is poor, tumor burden is very high, renal dose is limiting, or institutional infrastructure is unavailable. Future trials should therefore test radiometabolic therapy in settings where its mechanism is genuinely differentiated rather than as empirical late-line consolidation after patients have already declared aggressive disease biology.

The most mature combination strategy is radioimmunotherapy consolidation after induction therapy. In the phase III First-Line Indolent Trial, patients with advanced-stage follicular lymphoma who achieved complete response, unconfirmed complete response, or partial response after first-line induction were randomized to 90Y-ibritumomab tiuxetan consolidation or no further treatment. Among 414 enrolled patients, consolidation significantly prolonged median progression-free survival compared with observation, 36.5 versus 13.3 months, with a hazard ratio of 0.465. Benefit was observed both in patients achieving partial response after induction, with median progression-free survival of 29.3 versus 6.2 months, and in those achieving complete or unconfirmed complete response, with median progression-free survival of 53.9 versus 29.5 months. Importantly, 77% of patients in partial response after induction converted to complete or unconfirmed complete response after 90Y-ibritumomab tiuxetan, resulting in a final complete/unconfirmed complete response rate of 87% [6]. These data established radioimmunotherapy as an effective consolidation approach in chemosensitive follicular lymphoma, although subsequent long-term analyses did not show an overall survival advantage [7].

A similar principle has been explored in relapsed follicular lymphoma using abbreviated immunochemotherapy followed by 90Y-ibritumomab tiuxetan. Illidge et al. reported a phase II study in which patients with recurrent follicular lymphoma received three cycles of R-CHOP or R-CVP followed by 90Y-ibritumomab tiuxetan consolidation. Among 52 enrolled patients, 50 received radioimmunotherapy. The overall response rate after the full strategy was 98%, with complete or unconfirmed complete response in 30% and partial response in 68%. Eighteen patients converted from partial response after chemotherapy to complete/unconfirmed complete response after radioimmunotherapy, corresponding to a conversion rate of 40%. With a median follow-up of five years, median progression-free survival was 23.1 months and estimated five-year overall survival was 77.5%. The regimen was feasible, with manageable hematologic toxicity and grade 3–4 infective episodes in 13.5% of patients [41]. This study is relevant because it suggests that prior rituximab exposure does not necessarily prevent subsequent anti-CD20 radioimmunotherapy activity.

Radioimmunotherapy consolidation has also been tested in aggressive B-cell lymphoma, but the evidence is less consistent. In advanced-stage diffuse large B-cell lymphoma, the SWOG S0433 trial evaluated R-CHOP followed by additional CHOP and 131I-tositumomab consolidation. Eighty-four eligible patients were treated, but only 56 completed the full planned protocol. Among all evaluable patients, the objective response rate was 86%, and among those completing the full treatment course, 98% achieved partial or complete response. However, the two-year progression-free survival estimate was 69% and the two-year overall survival estimate was 77%, failing to meet the predefined threshold for further evaluation [42]. Early progression, deaths, toxicity, and declining performance status during chemotherapy limited the proportion of patients who could actually receive radioimmunotherapy consolidation. This study is important because it shows that late consolidation may be insufficient for biologically aggressive disease, especially when high-risk patients progress before receiving the radionuclide component.

In contrast, in limited-stage aggressive B-cell lymphoma with adverse risk features, ibritumomab consolidation after abbreviated chemotherapy and radiotherapy appeared feasible and produced favorable long-term outcomes. In SWOG S0313, 46 eligible patients received three cycles of CHOP followed by involved-field radiotherapy and 90Y-ibritumomab tiuxetan consolidation. With a median follow-up of 7.3 years, estimated progression-free survival was 89% at two years, 82% at five years, and 75% at seven years; estimated overall survival was 91%, 87%, and 82%, respectively. Toxicity was mainly hematologic, and no treatment-related myeloid neoplasms were observed [43]. Although this was a single-arm study with historical comparisons, it supports the feasibility of radioimmunotherapy consolidation when disease burden is limited and patients can complete the planned multimodality sequence.

Beyond CD20-directed strategies, CD22-directed radioimmunotherapy has been tested after frontline immunochemotherapy. In elderly patients with untreated diffuse large B-cell lymphoma, consolidation with fractionated 90Y-epratuzumab tetraxetan after R-CHOP showed potential activity but substantial hematologic toxicity. In this phase II trial, 71 patients received R-CHOP induction and 57 responders received 90Y-epratuzumab tetraxetan. The estimated two-year event-free survival was 75%. PET complete response increased from 56% after R-CHOP to 68% after radioimmunotherapy. However, grade 3–4 thrombocytopenia and neutropenia occurred in 84% and 79% of patients, respectively, and secondary myeloid neoplasms were reported [22]. Thus, CD22-directed consolidation remains biologically and clinically interesting, but toxicity and the absence of randomized validation limit immediate adoption.

A second major combination setting is transplantation. In this context, radionuclide-based therapy is not used simply as consolidation, but as part of conditioning or cytoreduction before hematopoietic stem cell support. CD45-directed radioimmunotherapy provides a distinct example. Because CD45 is expressed on most nucleated hematopoietic cells, it is unsuitable for conventional non-myeloablative treatment but attractive for conditioning. In a phase I study, 131I-labeled anti-CD45 antibody BC8 was administered with autologous stem cell support in 16 adults with relapsed, refractory, or high-risk lymphoma. Absorbed doses up to 32 Gy to the liver were delivered without dose-limiting toxicities. Among 14 patients with measurable disease, 57% achieved complete remission, including all three patients with T-cell non-Hodgkin lymphoma; three patients with B-cell non-Hodgkin lymphoma treated at the highest dose levels remained in complete remission without subsequent therapy at 35–41 months [27]. These findings support CD45-directed radionuclide therapy as a conditioning-associated strategy, although its use is necessarily restricted to specialized transplant settings.

CXCR4-directed radioligand therapy has also been explored mainly in transplantation workflows. In advanced diffuse large B-cell lymphoma, six heavily pretreated patients underwent ^68^Ga-pentixafor-based selection followed by CXCR4-directed radioligand therapy, conditioning chemotherapy, and allogeneic stem cell transplantation. The approach was feasible, and engraftment occurred after a median of 11 days. However, two patients died early from infectious complications, and among the four evaluable patients, responses were limited to two partial and two mixed responses. Median progression-free survival was 62 days and median overall survival was 76 days [9]. These results indicate feasibility but also emphasize the poor prognosis and high competing risks in heavily pretreated aggressive lymphoma.

The experience in T-cell lymphoma appears more promising, although it remains based on very small numbers. In four patients with advanced, heavily pretreated relapsed T-cell lymphoma, CXCR4-targeted radioligand therapy was used before allogeneic or autologous stem cell transplantation. Pretherapeutic dosimetry indicated lymphoma-absorbed doses up to 33.2 Gy. Engraftment was achieved in three patients; one partial response and two complete metabolic responses were reported among evaluable patients. Median progression-free survival was seven months, and after a median follow-up of 54 months, three patients were alive [10]. These findings suggest that CXCR4-directed therapy may have meaningful antitumor activity in selected T-cell lymphoma cases, but the evidence remains preliminary and highly selected.

The possible integration of radiometabolic therapy with immunotherapy, including immune checkpoint blockade, remains a forward-looking concept rather than an established clinical strategy in lymphoma. Radiation can theoretically promote immunogenic cell death, antigen release, inflammatory signaling, and microenvironmental remodeling, potentially enhancing immune-mediated tumor control. Conversely, severe lymphopenia, marrow suppression, and prior treatment burden may impair immune responsiveness and increase infectious risk. Therefore, combinations with checkpoint inhibitors, bispecific antibodies, antibody–drug conjugates, or cellular therapies should not be assumed to be automatically synergistic. In aggressive lymphoma, the negative or mixed experience of late radioimmunotherapy consolidation after prolonged induction suggests that timing is critical: for biologically high-risk disease, radionuclide-based approaches may need earlier integration, better molecular selection, or combination with agents selected according to disease biology rather than empirical addition at the end of therapy [42].

Overall, combination strategies show that radiometabolic therapy appears most defensible in biologically favorable settings: chemosensitive indolent lymphoma, low tumor burden, consolidation after response, or carefully selected transplantation protocols. In aggressive lymphomas, feasibility has been demonstrated, but efficacy is inconsistent and often limited by early progression before the radionuclide component can be delivered. Future studies should therefore focus on target expression, timing, tumor burden, marrow reserve, dosimetry, and rational combination with modern systemic or immune therapies.

Across combination strategies, the strongest evidence remains anti-CD20 RIT consolidation in chemosensitive follicular lymphoma. Abbreviated immunochemotherapy followed by RIT in relapsed follicular lymphoma and limited-stage aggressive B-cell lymphoma appears feasible but is supported mainly by phase II or historical-comparison data. CD22 consolidation after R-CHOP, CD45-based conditioning, and CXCR4-directed transplant-associated RLT remain exploratory and should be interpreted as specialized or trial-based strategies rather than routine treatment standards.

## 7. Practical and Implementation Issues

The historical experience of lymphoma radioimmunotherapy shows that clinical activity alone is insufficient to ensure adoption. Radiometabolic therapy requires dedicated infrastructure, trained personnel, radiation-safety procedures, radiopharmaceutical availability, multidisciplinary coordination, and appropriate reimbursement pathways. These requirements partly explain why effective agents such as 90Y-ibritumomab tiuxetan and ^131^I-tositumomab remained underused in routine hematology practice despite evidence of activity.

For established radioimmunotherapy, treatment delivery requires close coordination between hematology, nuclear medicine, radiopharmacy, medical physics, nursing, and radiation-safety personnel. The EANM procedure guideline for 90Y-ibritumomab tiuxetan emphasized that treatment should be performed only in facilities licensed for unsealed radioactive sources, with authorized staff, appropriate radiochemical labeling procedures, quality control, patient counseling, and structured follow-up [44]. Patient selection is also operationally demanding. Relevant baseline requirements include adequate blood counts, limited marrow involvement, review of prior chemotherapy or radiotherapy, pregnancy exclusion, renal and hepatic assessment, and estimation of life expectancy. These criteria are not merely administrative; they directly affect the risk of marrow suppression, infection, bleeding, and delayed recovery.

Modern radioligand therapy programs require an even broader institutional framework. Minimum requirements include a radioactive materials license, an authorized user, a radiation safety officer, nursing capacity, nuclear medicine technologists, medical physics support, pharmacy involvement, dedicated treatment space, access to appropriate imaging, and partner oncologists for referral and follow-up [45]. Clinical programs also require standardized referral pathways, multidisciplinary review, patient education, radiation-safety discharge procedures, waste-management protocols, authorization and billing workflows, and a defined strategy for post-treatment laboratory and imaging follow-up [45]. These needs are particularly relevant for lymphoma because many emerging approaches remain investigational, are delivered in highly selected populations, or are embedded within transplantation protocols.

Access remains a major limitation. Anti-CD20 radioimmunotherapy declined because of commercial withdrawal, limited availability, regulatory and reimbursement complexity, and competition from easier-to-deliver systemic therapies. Newer approaches, such as CXCR4-directed theranostics, are available only in specialized centers with PET-based target assessment, individualized dosimetry, radiopharmaceutical production or supply chains, and transplant integration. Therefore, implementation should not be considered a secondary issue but a central determinant of whether radiometabolic therapy can realistically enter clinical practice.

### Toxicity and Organ-Specific Complications

Hematologic toxicity is the dominant and most consistent adverse effect of lymphoma radioimmunotherapy and radioligand therapy. Neutropenia, thrombocytopenia, and lymphopenia are expected consequences of marrow irradiation, circulating radiopharmaceutical exposure, prior chemotherapy, and baseline marrow reserve. In the FIT trial, 90Y-ibritumomab tiuxetan consolidation was associated mainly with grade 3–4 hematologic toxicity, with grade 3–4 infections in 8% of treated patients [6]. In relapsed follicular lymphoma treated with abbreviated immunochemotherapy followed by 90Y-ibritumomab tiuxetan, grade 3–4 thrombocytopenia and neutropenia occurred in 38.5% and 36.5% of patients, respectively, and grade 3–4 infective episodes occurred in 13.5% [41]. Similar marrow-related toxicity was observed in aggressive lymphoma combination strategies [42,43]. These data indicate that marrow reserve, prior therapy, marrow involvement, and timing relative to chemotherapy are critical practical variables.

For antibody–radionuclide conjugates directed against CD22 or CD37, hematologic toxicity also remains dose-limiting. Consolidation with 90Y-epratuzumab tetraxetan after R-CHOP in elderly diffuse large B-cell lymphoma produced substantial grade 3–4 thrombocytopenia and neutropenia [22]. With 177Lu-lilotomab satetraxetan, reversible grade 3–4 neutropenia and thrombocytopenia were frequent, and dosimetry studies identified red marrow as a key dose-limiting compartment [25,26]. Therefore, future development of antibody-based radiometabolic therapy should not focus only on response rates, but also on marrow dosimetry, predosing strategies, tumor-to-marrow absorbed dose ratio, and patient selection.

Renal toxicity is especially relevant for CXCR4-directed radioligand therapy with pentixather. Dosimetric studies indicate that, in non-myeloablative settings, red marrow may be dose-limiting, whereas in myeloablative approaches with stem cell support, kidney dose becomes a major limiting factor [32]. In the toxicity analysis of CXCR4-directed endoradiotherapy before hematopoietic stem cell transplantation, 25 therapies were administered to 22 heavily pretreated patients. All patients developed cytopenias, but non-hematologic toxicity was generally limited. Only two acute kidney failure events were observed; one grade 3 acute kidney failure occurred in the context of tumor lysis syndrome and led to treatment discontinuation [34]. Nephroprotection is therefore essential. In this setting, amino acid coinfusion with L-arginine and L-lysine was used to reduce renal tubular retention of the peptide, following principles adopted from peptide receptor radionuclide therapy [34]. This reinforces the need for individualized dosimetry and renal-protection protocols when translating CXCR4-directed therapy beyond experimental settings.

Hepatic and splenic uptake should also be considered, particularly with antibody–radionuclide conjugates and CXCR4-directed agents. In 90Y-ibritumomab tiuxetan dosimetry, spleen and liver receive measurable absorbed doses, although these organs are not usually the main dose-limiting sites in standard non-myeloablative treatment [44]. For 177Lu-lilotomab satetraxetan, liver, spleen, kidneys, red marrow, and tumor were relevant compartments for biodistribution and dosimetry assessment [24]. In the CXCR4/pentixather toxicity series, mild transaminase or bilirubin increases were observed, but no grade ≥ 3 acute liver toxicity was reported [34]. These findings suggest that liver and spleen uptake is important for planning and monitoring, but hematologic and renal constraints are usually more clinically decisive.

Oral, dental, and salivary complications should be addressed carefully. In lymphoma radioimmunotherapy, clinically relevant salivary toxicity is not a dominant or well-established dose-limiting issue, unlike other radionuclide therapies in which salivary glands may receive higher radiation exposure. Nevertheless, oral and dental evaluation may be relevant in heavily pretreated patients, especially before myeloablative therapy or transplantation, where mucosal injury, infection risk, poor dentition, and neutropenia can interact clinically. Isolated symptoms such as dry mouth have been reported in multimodality lymphoma regimens including radioimmunotherapy, but available lymphoma studies do not support overemphasizing salivary toxicity as a major specific complication [43]. Therefore, the practical approach should be conservative: oral and dental status should be optimized before intensive or transplant-associated radionuclide strategies, but salivary toxicity should not be presented as a central limitation of lymphoma RIT/RLT unless supported by agent-specific data.

Secondary malignancies, myelodysplastic syndrome, and acute myeloid leukemia remain important long-term safety concerns. In the long-term FIT update, the annualized incidence of myelodysplastic syndrome or acute myeloid leukemia was higher after 90Y-ibritumomab tiuxetan consolidation than in the control arm [7]. In the randomized comparison of 90Y-ibritumomab tiuxetan consolidation versus rituximab maintenance, the cumulative incidence of second neoplasms at 10 years was higher in the radioimmunotherapy arm [18]. Conversely, some studies reported no treatment-related myeloid neoplasms during follow-up [16,17,42]. These apparently divergent findings likely reflect differences in baseline risk, prior therapies, follow-up duration, patient selection, and study size. Long-term hematologic surveillance should therefore be considered mandatory in future studies.

Clonal hematopoiesis provides an additional framework for interpreting these late events. In a retrospective cohort of 58 heavily pretreated follicular lymphoma patients receiving RIT, clonal hematopoiesis was frequent before RIT and increased during subsequent therapy exposure; patients who developed therapy-related myeloid neoplasms had higher clonal burden and greater clonal complexity [46]. More broadly, recent data outside lymphoma suggest that prior radiation therapy may be associated with increased risk of clonal hematopoiesis, particularly in relation to higher radiation exposure [47]. Direct causal attribution to lymphoma radiometabolic therapy remains uncertain, because prior chemotherapy, age, baseline marrow injury, and subsequent therapies are major confounders. Nevertheless, future trials should consider baseline and longitudinal assessment of clonal hematopoiesis when late myeloid risk is a key safety endpoint.

Infectious complications are particularly relevant in conditioning and transplantation-associated strategies. In CXCR4-directed endoradiotherapy before hematopoietic stem cell transplantation, grade 3–4 infections occurred after 9 of 25 treatment courses, although they were generally manageable and did not prevent subsequent conditioning and transplantation in most cases [34]. In the small DLBCL CXCR4-directed therapy series, early infectious deaths occurred in a heavily pretreated population [9]. Similar concerns were observed in T-cell lymphoma, where one patient died from septicemia before engraftment [10]. These findings do not necessarily indicate excessive agent-specific toxicity, but they highlight the narrow therapeutic window in profoundly immunocompromised patients.

Overall, practical implementation and toxicity are inseparable in lymphoma radiometabolic therapy. The most relevant risks are hematologic toxicity, marrow reserve impairment, infection, renal exposure in peptide-based CXCR4 therapy, and late secondary myeloid neoplasms. Liver, spleen, oral, dental, and salivary issues should be monitored, but their relevance depends strongly on the specific agent, administered activity, treatment setting, and patient condition. Future studies should include standardized toxicity reporting, baseline marrow and organ-risk stratification, individualized dosimetry when appropriate, and predefined supportive-care pathways.

## 8. Future Directions

Future development of radiometabolic therapy in lymphoma should be driven by target biology, quantitative imaging, dosimetry, and rational trial design rather than by empirical addition of radionuclides to existing treatment sequences. The historical experience with anti-CD20 radioimmunotherapy shows that efficacy is not sufficient if implementation is difficult, patient selection is suboptimal, or competing therapies are easier to deliver. Therefore, future strategies should focus on clinically meaningful niches where radiometabolic therapy has a plausible advantage: low-volume chemosensitive indolent lymphoma, target-positive relapsed or refractory disease, transplantation-associated cytoreduction, or biologically selected aggressive lymphoma.

A precision-theranostic workflow should combine molecular subtype, target-expression imaging, tumor burden, marrow reserve, renal and hepatic function, prior treatment history, clinical risk models, and dosimetric parameters. In this model, treatment allocation would be driven by the convergence of biological plausibility and deliverability: target-positive imaging alone would not be sufficient if absorbed dose is unlikely to be therapeutic or if organ/marrow constraints are prohibitive. Conversely, multi-omics biomarkers without actionable target expression would not justify radionuclide therapy. This integrated approach is summarized conceptually in Figure 3 and should be prospectively tested rather than assumed.

Alpha-emitting radionuclides represent one of the most interesting but still preliminary directions. Their high linear energy transfer and short path length may theoretically allow potent cytotoxicity with less dependence on long-range cross-fire radiation. This could be useful for microscopic disease, small-volume residual disease, or heterogeneous antigen expression. In lymphoma, the first-in-human experience with the 227Th-labeled anti-CD22 antibody BAY 1862864 showed feasibility in relapsed or refractory CD22-positive B-cell non-Hodgkin lymphoma, with an objective response rate of 25% and manageable toxicity, but without establishing clear clinical efficacy [23]. At present, alpha-emitting approaches should therefore be considered investigational. Their future role will depend on improved target selection, marrow-sparing strategies, pharmacokinetic optimization, and prospective evaluation in better-defined lymphoma populations.

Personalized dosimetry is another key direction. Fixed-activity approaches are simpler and were historically adopted for agents such as 90Y-ibritumomab tiuxetan, but emerging radioligand and antibody–radionuclide platforms show relevant interpatient variability in tumor uptake, marrow exposure, kidney dose, and organ biodistribution [32,34,44]. CXCR4-directed therapy illustrates this particularly well: in non-myeloablative settings, red marrow may be dose-limiting, whereas in myeloablative strategies with stem cell support, renal absorbed dose becomes a major constraint [32,34]. Individualized dosimetry may help define safe administered activity, optimize therapeutic index, and support rational integration with chemotherapy or transplantation. However, it also increases complexity, requires quantitative imaging, medical physics expertise, repeated acquisitions, and standardized methodology [45]. Therefore, dosimetry should be developed pragmatically, focusing first on settings where it is likely to change treatment decisions.

The main conceptual advance is integration between imaging and therapy. CXCR4 theranostics is the clearest example in lymphoma, because 68Ga-pentixafor PET can assess target expression and disease distribution before treatment with 177Lu- or 90Y-pentixather [9,10,29,30,31,32,33]. This creates a model in which PET is not only a diagnostic or response-assessment tool, but also a gatekeeper for radionuclide therapy. Future studies should define target-expression thresholds, account for interlesional heterogeneity, standardize PET interpretation, and determine whether imaging-derived metrics predict absorbed dose, response, toxicity, or survival. Without such validation, theranostic imaging risks remaining biologically plausible but clinically unvalidated.

Future trials should also be more selective. Broad unselected studies in aggressive lymphoma are unlikely to be successful if patients progress before receiving the radionuclide component. Earlier integration, molecular risk stratification, low-burden disease settings, and biomarker-selected enrollment may be more appropriate. Endpoints should include not only response rate and progression-free survival, but also treatment completion, marrow recovery, infectious complications, renal toxicity, late myeloid neoplasms, quality of life, and feasibility of implementation across centers. In this field, a clinically effective therapy that cannot be delivered reliably will not become a standard.

## 9. Conclusions

Radiometabolic therapy in lymphoma is a biologically sound but clinically uneven field. Established anti-CD20 radioimmunotherapy demonstrated meaningful activity, particularly in follicular lymphoma and selected indolent B-cell lymphomas, with high response rates, conversion from partial to complete response, and prolonged disease control in consolidation settings. However, lack of consistent overall survival benefit, hematologic toxicity, late safety concerns, logistical complexity, reimbursement issues, and commercial limitations prevented broad adoption.

Emerging approaches targeting CD22, CD37, CD45, and CXCR4 have renewed interest in the field. CD22- and CD37-directed antibody–radionuclide conjugates have produced clinically relevant responses, but hematologic toxicity remains a major limitation. CD45-directed therapy is best interpreted as a transplantation-conditioning strategy. CXCR4-directed theranostics is the most developed theranostic platform, linking PET-based target assessment with radioligand therapy, but current therapeutic evidence remains limited to small, highly selected cohorts.

Overall, radiometabolic therapy should not be presented as a broadly established treatment option for lymphoma outside specific historical indications or specialized investigational settings. Its future value will depend on precise target selection, imaging-based eligibility, individualized dosimetry, careful toxicity management, and integration into realistic multidisciplinary workflows. The most credible path forward is not a return to empiric radioimmunotherapy for all lymphoma patients, but a more selective theranostic model in which radionuclide therapy is used where its biological and clinical advantages are most likely to matter. In this sense, the field is better interpreted as a delayed and narrower opportunity than as a fully abandoned therapeutic concept.

## Figures and Tables

**Table 1:** Summary of established and emerging radiometabolic therapy strategies in lymphoma.

Strategy/Target	Representative Agents	Clinical Setting	Key Evidence	Guideline-Informed Current Interpretation
Anti-CD20 RIT	90Y-ibritumomab tiuxetan; 131I-tositumomab	Indolent B-cell lymphoma; relapsed/refractory disease; post-induction consolidation	High response rates; improved response depth; longer PFS/time to next treatment in consolidation; no consistent OS advantage [4,5,6,7,13,14,15,16,17,18,19]	Guideline-recognized niche option in selected indolent B-cell lymphoma; validated but rarely used because of toxicity, logistics, and availability [11,12].
CD22-directed RIT	90Y-epratuzumab tetraxetan; 227Th anti-CD22 antibody	Relapsed/refractory B-cell NHL; post-R-CHOP consolidation in elderly DLBCL	Meaningful activity in early-phase NHL studies; post-R-CHOP response deepening; early alpha-therapy feasibility [20,21,22,23]	Not NCCN/ESMO standard; investigational/clinical-trial strategy despite early clinical activity [11,12].
CD37-directed RIT	177Lu-lilotomab satetraxetan	Relapsed/refractory indolent NHL, especially follicular lymphoma	Clinically relevant responses, including rituximab-refractory disease; predosing improves tumor-to-marrow dosimetry [24,25,26]	Not NCCN/ESMO standard; investigational strategy supported mainly by phase 1/2 and dosimetry data [11,12].
CD45-targeted radionuclide therapy	131I-labeled anti-CD45 antibody (BC8)	High-risk lymphoma in transplant-associated conditioning	Feasible with autologous stem cell support; complete remissions reported in selected high-risk patients [27]	Not routine guideline-supported therapy; specialized transplantation-conditioning approach [11,12].
CXCR4 theranostics	68Ga-pentixafor PET + 177Lu/90Y-pentixather	Advanced target-positive lymphoma; transplant-associated cytoreduction/conditioning	PET enables in vivo target assessment; feasibility shown in DLBCL/T-cell lymphoma; dosimetry supports individualized planning [9,10,28,29,30,31,32,33,34]	Not NCCN/ESMO standard; early theranostic strategy for selected CXCR4-positive disease in trials or specialized centers [11,12].

Note: Current interpretation was anchored to contemporary NCCN and ESMO lymphoma recommendations. Only anti-CD20 radioimmunotherapy has a guideline-recognized niche in selected indolent B-cell lymphoma contexts; the other strategies summarized here are not considered standard-of-care lymphoma treatments by these guidelines and are therefore classified as investigational or specialized [11,12].

**Table 2:** Representative clinical evidence for established anti-CD20 radioimmunotherapy and consolidation strategies.

Study	Disease Setting	Strategy	Key Results	Interpretation
Witzig et al. [13]	Relapsed/refractory indolent or transformed B-cell NHL	90Y-ibritumomab tiuxetan vs. rituximab	ORR 80% vs. 56%; CR 30% vs. 16%	Improved response depth, but not clear TTP/DOR advantage
Witzig et al. [4]	Rituximab-refractory follicular NHL	90Y-ibritumomab tiuxetan	ORR 74%; CR 15%; median TTP 6.8 months	Proof of activity after rituximab failure
Kaminski et al. [5]	Chemotherapy-refractory low-grade/transformed NHL	131I-tositumomab	ORR 65%; CR 20%; longer DOR vs. prior chemotherapy	Established Bexxar activity in heavily pretreated disease
Kaminski et al. [15]	Previously untreated follicular lymphoma	131I-tositumomab	ORR 95%; CR 75%; median PFS 6.1 years	High activity, but single-arm study
FIT trial [6,7]	Advanced follicular lymphoma after induction response	90Y-ibritumomab tiuxetan consolidation	Median PFS improved; PR-to-CR/CRu conversion 77%; no OS advantage	Strongest randomized consolidation evidence
Alhaj Moustafa et al. [17]	Untreated low-grade FL/MZL	Single-agent 90Y-ibritumomab tiuxetan	ORR 100%; CR 94%; durable CRs in selected patients	Real-world support, but retrospective and selected
Lopez-Guillermo et al. [18]	Follicular lymphoma after R-CHOP response	90Y-ibritumomab vs. rituximab maintenance	10-year PFS 50% vs. 56%; higher second neoplasms in RIT arm	Less favorable versus modern maintenance
Puvvada et al. [19]	Relapsed follicular lymphoma after ESHAP	Cytoreduction followed by 90Y-ibritumomab	ORR 72%; CR 45%; median PFS 10 months	Demonstrates limits in high-risk relapse

## Data Availability

No new data were created or analyzed in this study. Data sharing is not applicable to this article.

## Abbreviations

The following abbreviations are used in this manuscript:

AML	acute myeloid leukemia
ASCT	autologous stem cell transplantation
CR	complete response
CXCR4	C-X-C chemokine receptor type 4
DLBCL	diffuse large B-cell lymphoma
EFS	event-free survival
FDG	fluorodeoxyglucose
FL	follicular lymphoma
HSCT	hematopoietic stem cell transplantation
MDS	myelodysplastic syndrome
NHL	non-Hodgkin lymphoma
ORR	overall response rate
PET	positron emission tomography
PFS	progression-free survival
RIT	radioimmunotherapy
RLT	radioligand therapy
CAR-T	chimeric antigen receptor T-cell therapy
CH	clonal hematopoiesis
CHIP	clonal hematopoiesis of indeterminate potential
